# Supplementary material for: Characterization of polarization states of canine monocyte derived macrophages
Source: PLoS One. 2023 Nov 8;18(11):e0292757. doi: 10.1371/journal.pone.0292757 (PMC10631683; doi:10.1371/journal.pone.0292757)
Supplement: S1 Fig — (PDF) [file pone.0292757.s004.pdf]

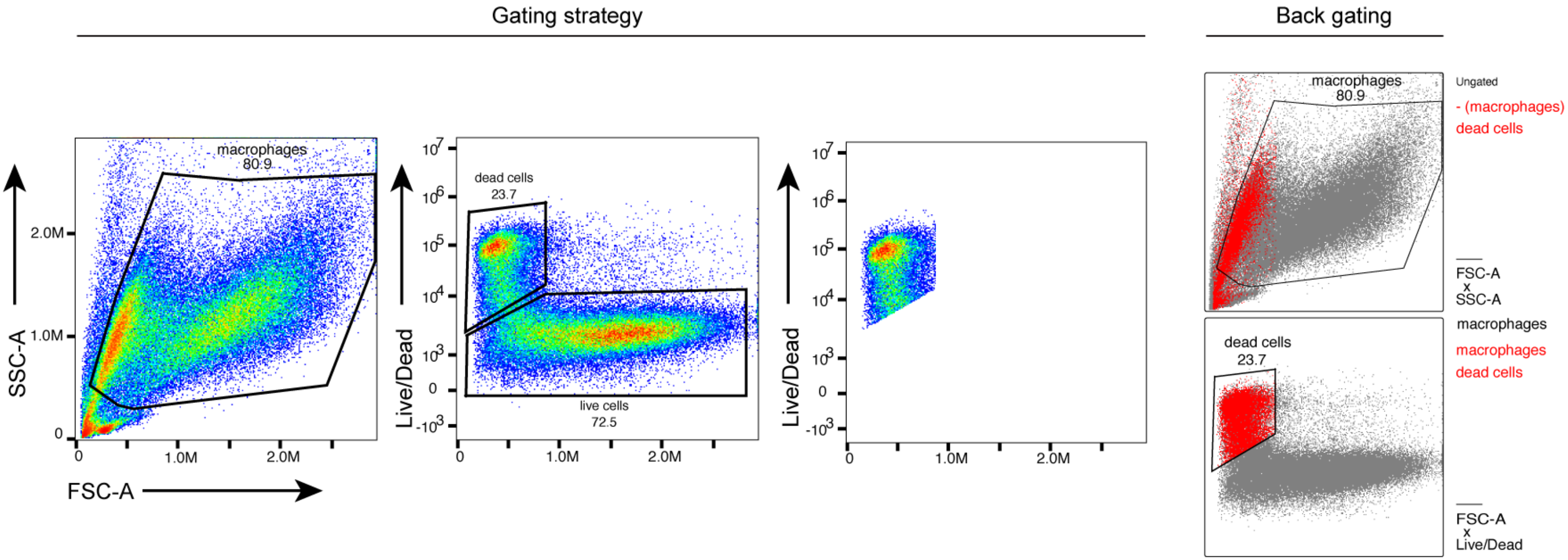

Fig. S1. Identification of non-viable cells by back gating. Polarized canine MDMs (M1 and M2) and undifferentiated M0 cells were collected on day 7, stained with ViaKrome Fixable Viability Dyes and analyzed by flow cytometry. To exclude dead cells, back gating was performed using FlowJo 10.8.2. identified dead cells were excluded in FSC/SSC panel in all experiments.
