# Supplementary figures and images for: Characterization of polarization states of canine monocyte derived macrophages
Source: PLoS One. 2023 Nov 8;18(11):e0292757. doi: 10.1371/journal.pone.0292757 (PMC10631683; doi:10.1371/journal.pone.0292757)

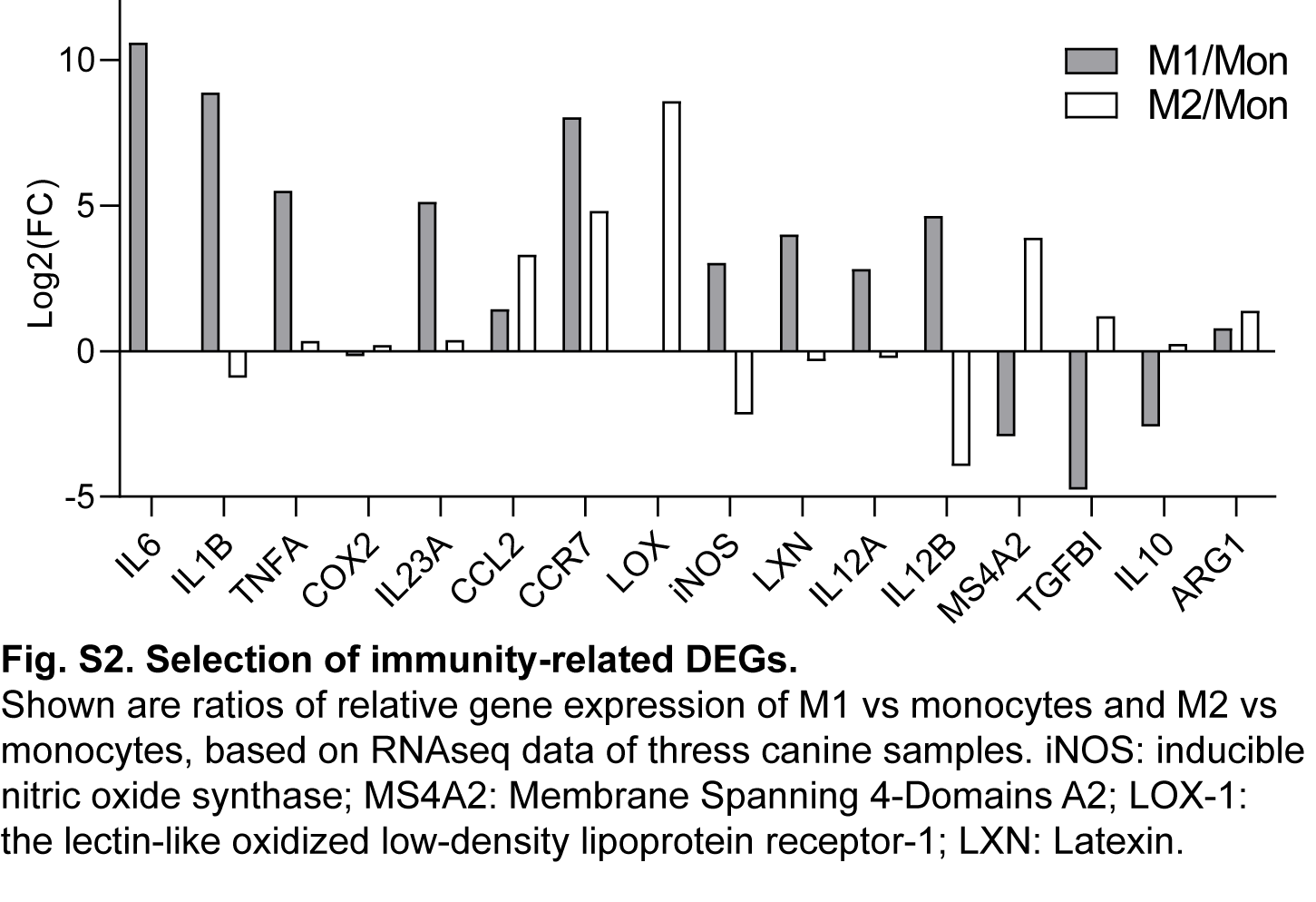

Supplement: S2 Fig — (TIF) [file pone.0292757.s005.tif]
