# Supplementary material for: Characterization of polarization states of canine monocyte derived macrophages
Source: PLoS One. 2023 Nov 8;18(11):e0292757. doi: 10.1371/journal.pone.0292757 (PMC10631683; doi:10.1371/journal.pone.0292757)
Supplement: Attachment — Submitted filename: Lyu et al response to reviewers.docx [file pone.0292757.s007.docx]

**editorial remarks**
Minor concerns
**1.** American English may be appropriate. For example, “RNAseq of the three macrophage subsets showed distinct gene expression profiles, which are closely associated with immune responsiveness, cell differentiation and phagocytosis.”

*Reply: We corrected the text to American English where appropriate.*

**2.** In the abstract, “M2- polarized MDMs” suddenly appeared. Please show elaboration of MDM at first appearance.

*Reply: We removed MDMs from the abstract. In the manuscript itself it is now described as monocyte-derived macrophages upon its first appearance Line 116.*

**3.** I think PLOS ONE does not ask to add keywords on the title page. Also, figure legends and a table should be in the result section. Please check the guideline.
<https://journals.plos.org/plosone/s/submission-guidelines>

*Reply: Keywords have been removed and the figure legends and table have been moved to the correct position in the manuscript text.*

**4.** In Figure 3A, the numbers in the Venn diagrams seem too small. Could you change them to larger for readers? This issue is the same for Figures 4 and 5.

*Reply: We have changed the fonts in Fig 3- 5 to make them easier to read.*

**Reviewer #1:** 
The manuscript "Characterization of polarization states of canine monocyte-derived macrophages" is well-written and experimentally well-conducted.
Canine macrophage polarization still has a poor level of scientific understanding. In this study, the authors polarized canine monocytes toward M1 and M2 macrophages and contrasted them to unpolarized M0 cells. The authors characterized polarized M1 and M2 and unpolarized M0 macrophages by morphology, gene profiles, surface marker features, and functional properties. The following analysis revealed that canine macrophages M1, M2, and M0 differ from one another and can be identified based on their phenotypic characteristics and the expression of particular markers, just like macrophages from other well-studied species. RNAseq revealed the three macrophage subsets' gene expression patterns closely related to phagocytosis, cell differentiation, and immune responsiveness. No new polarization markers could, however, be assigned by the authors.
Future research on (polarized) canine macrophage studies will benefit significantly from monocyte-derived polarized macrophages, particularly in studies of immune-related diseases in dogs. The differentiation protocols produced two subsets of macrophages (M1 and M2) that differ significantly in morphology, gene and protein expression, and functionality. These subsets are also a great model for future research on macrophage studies in general.
Recommendations.
The manuscript is accepted with minor revisions.
**1)** Discussion: add more biological information about DEGs found in M1 and M2 macrophages.

*Reply: We have added several lines on the biological information in the discussion. Line 481-484.*

**2)** Add more information regarding the significance of the DEGs specific for each subset of macrophages.

*Reply: From the RNAseq data we selected gene expression levels of some described M1 or M2 markers. This shows that indeed, for example Il-6 and IL-1β are much higher in M1 compared to M2 while TGF- β is higher in M2. Surprisingly arginase expression does not show major differences. We added this as a supplementary figure to the manuscript and describe the results in lines 358-363.*

**Reviewer #2:** Dear,
**1)** The authors explored an interesting research field and the main goal of the manuscript is really necessary to understand the macrophage polarization for canine monocytes. The authors proposed a protocol to characterize the macrophage polarization, and the main approaches were considered but it should be improved. The major concern is the basic experimental design to induce the macrophage polarization because the authors called M0 macrophages a cell type that was not differentiated to macrophage and is a monocyte.

*Reply: We thanks the reviewer for the nice remarks and indeed agree that M0 are not macrophages. We have now changed the terminology throughout the whole manuscript to either M0 monocytes or M0 cells.*

*However, regarding the 1^st^ remark about the basic design, (and this will come back in the rebuttal below), we feel that our aim of the manuscript was not clearly brought across. We do not seek to optimize the protocol to make polarized canine macrophages, but instead we want to characterize the polarized macrophages that are obtained using a standardized method. This method is well described in human and mouse, and recently a very small number of canine studies also used this protocol based on GM-CSF/IFNγ/LPS and M-CSF/IL-4. Finding the best protocol is a completely different study requiring a completely different set-up. In addition, we would not know how to clearly define ‘an improved method’ because it would be based on human and mouse macrophage readouts and would deny possible species differences in macrophage polarization and functionality. We added a few sentences in the discussion to emphasize this aim of our study again. Line 506-511.*

**2)** Moreover, the authors should demonstrated that most of the results found were not already induced by M/GM-CSF, then control groups containing only M-CSF or GM-CSF is strongly recommended.

*Reply: We performed these experiments as requested and polarized M0 cells towards M1/M2 using either GM-CSF / M-CSF alone, or with the addition IFN-γ/LPS and IL-4. We performed qPCR on specific M1 and M2 markers and also checked the cell’s morphology. These experiments showed that indeed the presence of the growth factors alone already had strong polarizing activity, as seen by the morphology, see Figure E(xtra)1 below . However, the ‘extra’ addition of IFN-γ / LPS resulted in an upregulation of IL-6 (M1 marker) compared to GM-CSF alone, while addition of IL-4 induced gene expression of TGF-beta and MS4A2 (M2 marker), showing that these factors have an additional effect on gene expression of polarization marker genes (Fig. E2). These actual experiments are, however outside the scope of the manuscript. We have included a comment on our findings in the discussion. Line 532-536.*

*Fig E1. Morphology of M1 and M2 macrophages on day 8 after polarization. Polarization with GM-CSF with or without IFNγ/LPS resulted in a M1 phenotype. Polarization with M-CSF with or without IL-4 resulted in a M2 phenotype.*

*Fig. E2. Relative gene expression of cells matured in the presence of A) GM-CSF ± IFN/LPS and B) M-CSF ± IL-4.*

**3)** LPS can also induce some characteristics related to the M2 macrophages for murine and human cells in a dose-dependent manner, then it should be discussed by the authors or demonstrate that 100ng/mL of LPS is adequate to evaluate only M1 macrophage.

*Reply: We agree with the reviewer that LPS indeed can cause some M2 characteristics. Before performing our study on monocyte derived macrophages we did some initial studies on the effect on LPS on canine macrophages in the macrophage cell line 030D which indicated that this concentration indeed induced classical M1 markers. Eventually, the results of our monocyte derived macrophages polarization also shows that this methodology indeed results in M1 like features.*

**4)** The association of IL-12 and IFN-g could be a better option for M1 macrophage induction. So, the impact of LPS/IFN and IL-4 in the macrophage polarization is confused with M/GM-CSF and so far there is no M0 macrophage.

*Reply: We agree with the reviewer that several variables in the polarization methodology have not been explored, including the use of IL-12 and IFNy. However, as stated above, the focus of this study is not to optimize the polarization method.*

*We agree with the wrong use of the term M0 macrophages and this has been replaced throughout the manuscript.*

**5)** In addition, iNOs and Arginase-1 were missed and must be evaluated to complement the flow cytometry data, and both mRNA relative expression and protein expression are well accepted.

*Reply: We have added the gene expression of several key genes in supplementary Figure S1 , including iNOS and Arginase1 and discuss these in the manuscript, line 358-363. Unfortunately we don’t have assays running for determination of these enzymes on a protein level (much less tools are available for canine research than mouse and human).*

**6)** The morphological analysis should be improved, and H&E staining is an example for that.

*Reply: Unfortunately , we made an error in the morphological analyses of our polarized macrophages in our new set of experiments, and only stained with Hematoxylin. We added these stainings below for your convenience but realize they don’t add any value. However, we are not completely sure what the reviewer would like to see with a correct H&E staining. If it is essential, we are willing to obtain fresh canine blood, polarize monocytes to macrophages and perform the analysis. But considering the infrequency of obtaining fresh buffy coats from (canine) patients we doubt if the extra effort is really required.*

**7)** The phagocytosis assay could consider the incubation with IFN-g alone for each condition as a positive control to facilitate the engulfment of beads.

*Reply: We agree that many variables can be tested to optimize phagocytosis or other characteristics of polarized macrophages. But this falls out of the scope of the current manuscript which is a characterization of in vitro M1 and M2 polarized macrophages in dogs. The contribution of each polarization-inducing compound is subject to a follow up study.*

**8)** The plasticity of canine monocytes should be validated by the repolarization assay, and the balance between iNOS/Arginase-1 is a great parameter to demonstrate that M1/M2 subsets can be repolarized.

*Reply: In our extra set of experiments of experiments we used our day8 M1 and M2 polarized macrophages and treated them with the opposite polarizing environments. So M2 polarized macrophages were grown for an additional 2 days in the presence of GM-CSF/ IFN-γ and LPS and day 8 M1 polarized macrophages were grown an additional 2 days in the presence of M-CSF and Il-4. Control M1 and M2 macrophages were grown an additional 2 days in their original growth medium. Clear morphological changes were observed for M2🡪 M1 repolarization (Fig. E3), and also gene expression of IL-6, was upregulated (Fig. E4). The M1🡪M2 repolarization was more subtle in both aspects. Morphological changes were less clear, and we didn’t see the typical M2-like morphology. However, on a gene level a tendency towards more MS4A2 (M2 marker) gene expression was observed. Based on these results we can conclude that at day 8 both M1 and M2 had not reached an end-point polarization state and still had plasticity. However, we cannot state that they repolarized to classical M1 or M2 macrophages. We made a comment about the importance of repolarization in the discussion in Line 532-536. We will pursue the re-polarization study in our planned follow up studies, also optimizing this repolarization protocol.*

*Fig. E3. Morphology of repolarized macrophages on day 10 after (re-)polarization. Day 8 M1 macrophages did not clearly change morphology after 2 days repolarization with M2 conditions (top left vs top right image). M2 macrophages on the other hand clearly depicted a mor eM1-like morphology after repolarization in M1 conditions (compare bottom left and bottom right image.**Fig. E4. Relative gene expression of cells matured for 8 days towards M1 or M2, and on day 9 repolarized towards opposite polarization state.*

6. PLOS authors have the option to publish the peer review history of their article ([what does this mean?](https://journals.plos.org/plosone/s/editorial-and-peer-review-process#loc-peer-review-history)). If published, this will include your full peer review and any attached files.


If you choose “no”, your identity will remain anonymous but your review may still be made public.
