# Supplementary material for: Characterization of polarization states of canine monocyte derived macrophages
Source: PLoS One. 2023 Nov 8;18(11):e0292757. doi: 10.1371/journal.pone.0292757 (PMC10631683; doi:10.1371/journal.pone.0292757)
Supplement: Attachment — Submitted filename: point to point reply.docx [file pone.0292757.s008.docx]

**Characterization of polarization states of canine monocyte derived macrophages.**

Qingkang Lyu, Edwin J.A. Veldhuizen , Irene S. Ludwig, Victor P. M. G. Rutten, Willem van Eden, Alice J. A. M. Sijts and Femke Broere

**Point to point reply.**

***Comments to the Author***
Reviewer #3: Overall, the authors have thoroughly addressed the majority of the reviewer comments. However, there are still a few items that require further clarification.


1. New reviewer comment: It’s the reviewer’s understanding that based on the manuscript changes the authors meant “…read counts lower than 2 in all samples…” rather than “2 fold difference”, please affirm whether this is true. If 2 fold difference was intended, please edit the manuscript accordingly.

*We confirm that this is true. Genes with <2 counts for all samples, were not taken along in the determination which genes were differentially expressed. No changes made to the manuscript.*

2. new reviewer comment: The back-gating data was not included in the submission (or was not visible to the reviewer), therefore could not be verified. It is suggested that a supplemental figure with wider gating showing the live vs. dead population be added.

*We have now added the figure showing back-gating as a supplementary file (Fig S1) and referred to it in the text in Line 275 of the revised manuscript.*

3. Figure resolution should be addressed upon final figure submission to ensure readability.

*We checked figure resolution of all figures and they are all >300 dpi.*
